# Evolution of the interaction between Runx2 and VDR, two transcription factors involved in osteoblastogenesis

**DOI:** 10.1186/1471-2148-10-78

**Published:** 2010-03-17

**Authors:** Sylvain Marcellini, Carola Bruna, Juan P Henríquez, Miguel Albistur, Ariel E Reyes, Elias H Barriga, Berta Henríquez, Martín Montecino

**Affiliations:** 1 Departamento de Biología Celular, Facultad de Ciencias Biológicas, Universidad de Concepción, Casilla 160-C, Concepción, Chile; 2 Departamento de Bioquímica y Biología Molecular, Facultad de Ciencias Biológicas, Universidad de Concepción, Casilla 160-C, Concepción, Chile; 3 Facultad de Ciencias de la Salud, Universidad Diego Portales, Av Ejército 141, piso 4, Santiago, Chile; 4 Current address: Laboratorio de Biología del Desarrollo, Facultad de Ciencias Biológicas, Universidad Andrés Bello, Av República 217, Piso 3, Santiago, Chile

## Abstract

**Background:**

The mineralized skeleton is a major evolutionary novelty that has contributed to the impressive morphological diversifications of the vertebrates. Essential to bone biology is the solidified extracellular matrix secreted by highly specialized cells, the osteoblasts. We now have a rather complete view of the events underlying osteogenesis, from a cellular, molecular, genetic, and epigenetic perspective. Because this knowledge is still largely restricted to mammals, it is difficult, if not impossible, to deduce the evolutionary history of the regulatory network involved in osteoblasts specification and differentiation. In this study, we focused on the transcriptional regulators Runx2 and VDR (the Vitamin D Receptor) that, in mammals, directly interact together and stabilize complexes of co-activators and chromatin remodellers, thereby allowing the transcriptional activation of target genes involved in extracellular matrix mineralization. Using a combination of functional, biochemical, and histological approaches, we have asked if the interaction observed between Runx2 and VDR represents a recent mammalian innovation, or if it results from more ancient changes that have occurred deep in the vertebrate lineage.

**Results:**

Using immunohistochemistry and *in situ *hybridization in developing embryos of chick, frog and teleost fishes, we have revealed that the co-expression of Runx2 and VDR in skeletal elements has been particularly strengthened in the lineage leading to amniotes. We show that the teleost Runx2 orthologue as well as the three mammalian Runx1, Runx2 and Runx3 paralogues are able to co-immunoprecipitate with the VDR protein present in nuclear extracts of rat osteoblasts stimulated with 1α,25-dihydroxyvitamin D_3_. In addition, the teleost Runx2 can activate the transcription of the mammalian *osteocalcin *promoter in transfection experiments, and this response can be further enhanced by 1α,25-dihydroxyvitamin D_3_. Finally, using pull-down experiments between recombinant proteins, we show that the VDR homologue from teleosts, but not from ascidians, is able to directly interact with the mammalian Runx2 homologue.

**Conclusions:**

We propose an evolutionary scenario for the assembly of the molecular machinery involving Runx2 and VDR in vertebrates. In the last common ancestor of actinopterygians and sacropterygians, the three Runx paralogues possessed the potential to physically and functionally interact with the VDR protein. Therefore, 1α,25-dihydroxyvitamin D_3 _might have been able to modulate the transcriptional activity of Runx1, Runx2 or Runx3 in the tissues expressing VDR. After the split from amphibians, in the lineage leading to amniotes, Runx2 and VDR became robustly co-expressed in developing skeletal elements, and their regulatory interaction was incorporated in the genetic program involved in the specification and differentiation of osteoblasts.

## Background

The formation and maintenance of the skeleton involves the specification and differentiation of specialized cell types, such as the osteoblasts, chondrocytes and osteoclasts [1-3]. The osteoblasts, of mesenchymal origin, are responsible for depositing the mineralized extracellular matrix of the bones. Their proliferation, survival, and physiology depend on a complex interplay between intrinsic and extrinsic signals. For example, if osteoblasts receive too few BMP signals, or if they fail to express the *Runx2 *and *osterix *transcription factors, their ability to differentiate into osteoblasts is largely compromised or abolished [4-6]. Runx2 was the first transcription factor identified as being essential for osteoblastogenesis [5,7]. It was later shown to act redundantly with its paralogue Runx3 during chondrogenesis [8]. Runx2 can either activate or repress transcription, depending on the nature of its cofactors, and on the regulatory architecture of the target promoter [Reviewed in [9-11]]. Although Runx2 has mainly been studied in mammals, we know that it is also expressed in chondrocytes and osteoblasts during the skeletogenesis of birds, frogs, teleost fishes and sharks [12-17]. In addition, morpholino knock-down approaches have demonstrated that Runx2 is required during chondrogenesis of *Danio rerio *and *Xenopus tropicalis *[14,18]. Taken together, these studies reveal a broad conservation of Runx2 function throughout osteichthyan vertebrates, a monophyletic group consisting of actinopterygians (such as teleost fishes) and sarcopterygians (such as tetrapods)[19].

Mammalian osteoblast specification and differentiation also rely on the activity of other transcription factors such as the Vitamin D Receptor (VDR) that belongs to the NR1I family of nuclear receptors [20,21]. The VDR is required for normal bone formation and regulates the transcription of target genes upon binding to its ligand, the 1α,25-dihydroxyvitamin D_3 _[22-25]. In the intestine, VDR and 1α,25-dihydroxyvitamin D_3 _play a pivotal role in the maintenance of bone mineralization and skeletal development through the regulation of proper calcium and phosphate absorption [26,27]. Importantly, the cell-autonomous contribution of *VDR *to osteoblastic differentiation has been demonstrated by several additional lines of evidence. First, the 1α,25-dihydroxyvitamin D_3 _enhances osteoblastic differentiation and stimulates the expression of VDR target genes coding for bone matrix components [28]. Second, osteoblasts express the Vitamin D receptor and also have the ability to synthesize 1α,25-dihydroxyvitamin D_3 _[24,29]. Third, cultures of primary osteoblasts harvested from a *VDR *knock-out mouse clearly exhibit a reduced mineralization potential [30]. Finally, the direct binding of the VDR to the promoters of osteoblast-specific genes like *osteocalcin *(*ocn*) and *bone sialoprotein *(*bsp*) is required for chromatin remodeling and transcriptional activation induced by 1α,25-dihydroxyvitamin D_3 _[31-33]. In this respect, it is relevant to note that the direct protein-protein interaction between VDR and Runx2 stabilizes transcriptional complexes at specific promoters, thereby contributing to osteogenesis [32]. In summary, a variety of experiments performed in adult tissues or in cell cultures have established VDR as an important osteoblast-specific transcription factor. However, the scarcity of developmental data and comparative studies clearly impede our understanding of the evolutionary mechanisms through which VDR became incorporated in the vertebrate skeletogenic regulatory network.

Indeed, changes in the regulatory interactions involved in cell specification and differentiation lie at the heart of the evolutionary process [34-36]. The functional experiments performed in mammalian osteoblasts have provided a detailed picture of the Runx2-dependent regulatory network supporting skeletogenesis. They revealed how the function of Runx2 is regulated at the transcriptional and post-translational level, and how distinct inputs converge on the Runx2 target genes. Yet, the lack of data from non-mammalian vertebrates renders difficult, if not impossible, to decipher how this skeletogenetic regulatory network emerged and evolved. In the present work, we focused our attention on a specific node of this complex network. In order to shed light on the evolutionary origin of the Runx2-VDR physical and functional interaction in osteoblasts, we have compared the function, the biochemical properties, and the expression patterns of their homologues from distantly related chordates. Based on our results, we propose that the three Runx paralogues and VDR had the potential to functionally and physically interact in the last common ancestor of osteichthyans, and most probably did so in tissues where both proteins were expressed, in the presence of 1α,25-dihydroxyvitamin D_3_. After the split from amphibians, in the lineage leading to amniotes, Runx2 and VDR became robustly co-expressed in developing skeletal elements, and their regulatory interaction was incorporated in the genetic program involved in the specification and differentiation of osteoblasts.

## Results

Several studies performed in chondrichthyans and a variety of osteichthyans have demonstrated that the *Runx2 *expression in developing skeletal elements is highly conserved [12-17]. The evolution of the *VDR *expression pattern is, however, less clear. The *VDR *transcripts are known to be fairly ubiquitous in lamprey, teleost, and frog adult tissues analyzed by RT-PCR or Northern blot [37]. Because few studies have described the spatio-temporal expression pattern of *VDR *during embryonic development [38], we decided to assess the degree of co-expression of Runx2 and VDR during skeletogenesis of distantly related oteichthyan vertebrate such as birds, amphibians and teleosts.

### VDR is robustly co-expressed with Runx2 in the osteoblastic lineage of amniotes but not of amphibians

**Figure 1:**
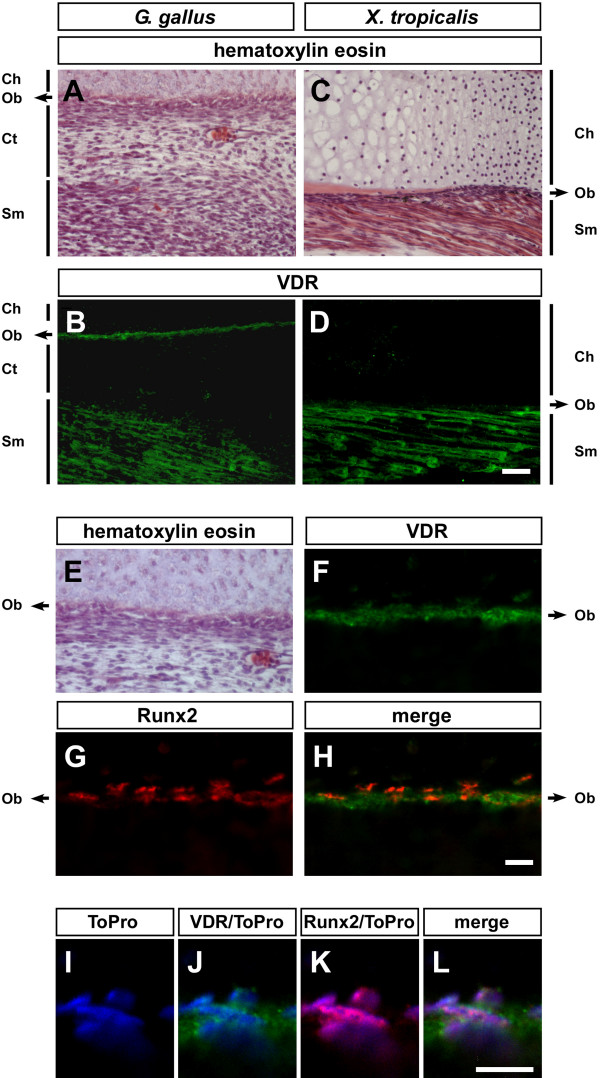
**Runx2 and VDR are co-expressed in the osteoblastic lineage in amniotes but not in amphibians**. The figure shows longitudinal sections of hindlimbs from E7 *Gallus gallus *(A-B, E-L) or from NF58 *Xenopus tropicalis *(C-D) specimens. (**A**) Hematoxilin staining showing the preosteoblasts of the developing perichondrium surrounded by hypertrophic chondrocytes, connective tissue and skeletal muscles. (**B**) Localization of the VDR protein in the skeletal muscles and cells of the osteoblastic lineage. (**C**) Hematoxilin staining showing the osteoblasts of the developing periosteum surrounded by hypertrophic chondrocytes and skeletal muscles. (**D**) Localization of the VDR protein in the skeletal muscles. (**E**) Hematoxilin staining of the perichondrium. (**F-H**) Fluorescent immunohistochemistry showing the localization of VDR (F), Runx2 (G) and the colocalization of both proteins (H) in the perichondrium. (**I-L**) Higher magnification of the perichondrium cells. Nuclei are visualized using ToPro either alone (I, blue channel), with VDR (J, green channel), Runx2 (K, red channel), or both proteins (L). Abbreviations: Ch, hypertrophic chondrocytes; Ob, cells of the osteoblastic lineage; Ct, connective tissue; Sm, skeletal muscles. Scale bars represent 25 micrometers in a-d; and 10 micrometers in e-h and i-l.

The fact that cell cultures of chick calvaria show VDR expression before the onset of typical osteoblastic markers suggests that VDR could be co-expressed with Runx2 in the nuclei of osteoblastic precursors before cell differentiation and matrix mineralization [39]. To verify this possibility, we assessed the co-expression of Runx2 and VDR in developing skeletal elements of *Gallus gallus *at embryonic day (E)7 (Fig. 1a). We detected the VDR protein in the preosteoblastst present in the perichondrium as well as in the striated muscles, a situation reminiscent to what has been described in mammals (Fig. 1b) [40]. Therefore, VDR seems to be robustly expressed in the osteoblastic lineage of both intramembraneous and endochondral chick bones. To demonstrate the high degree of co-expression of the VDR and Runx2 proteins, we examined double stainings by confocal microscopy and observed that chick preosteoblasts are significantly enriched in both proteins (Fig. 1 e-h). We further show that in developing chick skeletal elements Runx2 is strictly nuclear, while the VDR protein is evenly distributed in the cytoplasm and the nucleus, as expected (Fig. 1 i-l).

Because birds are closely related to mammals, we next whished to analyze the expression of VDR in a tertrapod species that does not belong to the amniota. For this purpose, we analyzed endochondral bone of the amphibian *Xenopus tropicalis *at stage NF58 (Fig. 1c). Immunohistochemical staining revealed a strong expression in the hindlimb skeletal muscles, showing that the VDR-specific antibody can efficiently recognize the *Xenopus *epitope (Fig. 1d). However, in sharp contrast with the situation observed with chick embryos, no specific signal could be observed in differentiating osteoblasts of the periosteum (Fig. 1d). Although we cannot rule out the possibility that the VDR is expressed below detection level in frog osteoblasts, the strong positive reaction the skeletal muscle of *Gallus *and *Xenopus *supports the idea that, in amphibians, the VDR protein is not present in developing endochondral bones (compare Fig. 1b and 1d). Because the presence of mRNA coding for Runx2 has already been demonstrated in the perichondrium and periosteum of several amphibian species [15,41,42], the absence of VDR in these tissues rules out the possibility of a functional interaction between the two proteins.

### The teleost VDR and Runx2 are co-expressed in some skeletal elements

**Figure 2:**
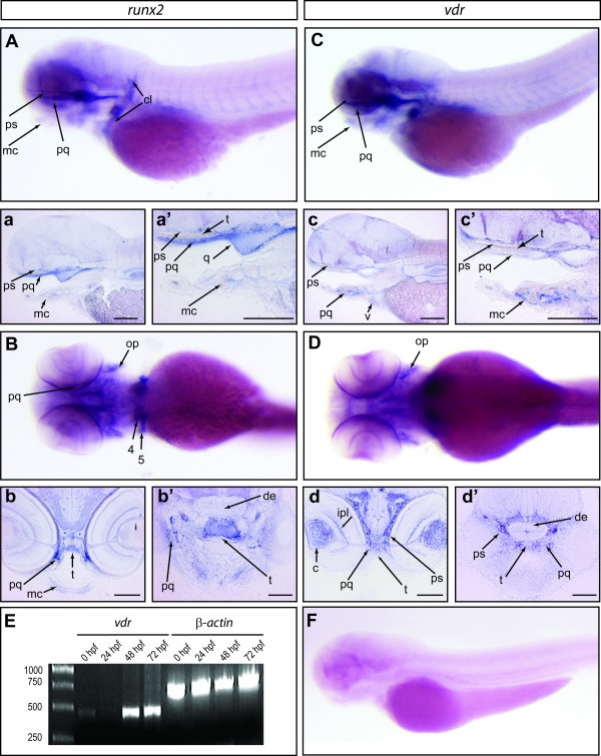
**Expression pattern of *Runx2b *and *VDR *in *Danio rerio***. An *in situ *hybridization of a 72 hpf zebrafish embryo hybridized with a *Runx2b *antisense probe is shown in a lateral (**A**) and ventral (**B**) view. The images in **a **and **a' **are sagittal sections of the specimen shown in **A**. The images in **b **and **b' **are transversal sections of the specimen shown in **B**. An in situhybridization of a 72 hpf zebrafish embryo hybridized with a *VDR *antisense probe is shown in a lateral (**C**) and ventral (**D**) view. The images in **c **and **c' **are sagittal sections of the specimen shown in **C**. The images in **d **and **d' **are transversal sections of the specimen shown in **D**. (**E**) RT-PCR performed on cDNA from zebrafish embryos at 0, 24 hpf, 48 hpf and larvae at 72 hpf with primers specific for *VDR *and β*-actin*. The molecular weight standard is shown on the left. (**F**) Lateral view of a 72 hpf zebrafish larvae hybridized with a *VDR *sense probe. Abbreviations: c, crystalline; cl, cleithrum; de, diencephalon; ipl, inner plexiform layer; mc, meckel cartilage; op, operculum; pq, palate quadrate; ps, parasphenoid; q, quadrate; t, trabecula; v, ventricle; 4 and 5, the IV and V branchial cartilage territories, respectively. The scale bar in a, a', b, b', c, c', d and d' represents 50 micrometers.

In order to examine if *Runx2 *and *VDR *are co-expressed to some degree in developing skeletal elements of teleosts, we analyzed embryos from *Danio rerio *(Fig. 2). We first used RT-PCR to describe the temporal expression pattern of the *Danio VDR *gene, and revealed a maternal expression, the absence of transcripts in 24 hours post fertilization (hpf) larvae, and the presence of zygotic transcripts from 48 hpf onward (Fig. 2e). We next compared the expression pattern of *Runx2 *and *VDR *in 72 hpf larvae because at this stage both genes are expressed, and skeletal elements are actively developing [43,44]. As a result of a genomic duplication event that occurred in the teleost lineage, *Danio rerio *possesses two highly conserved *Runx2 *paralogues, named *Runx2a *and *Runx2b *[12]. We employed an *in situ *hybridization probe containing the complete open reading frame of *Runx2b *that is likely to also anneal to *Runx2a *transcripts [12]. In this respect, it is important to stress out that at 72 hpf the expression pattern of *Runx2a *only represents a fraction of the *Runx2b *positive cells [12]. We found that the expression patterns of *Runx2b *and *VDR *differed in several respects. For instance, at this stage (72 hpf), *Runx2b *but not *VDR *transcripts are detected in the cleithrum, the fourth and fith branchial arches and quadrate (Fig. 2). Conversely, *VDR *but not *Runx2b *transcripts are detected in the inner plexiform layer (eye) and ventricle (heart) (Fig. 2). Most importantly, we also detected the presence of both mRNAs in several bones of the skull, such as the Meckel Cartilage (Lower Jaw), parasphenoid, palatoquadrate (Upper Jaw), and operculum (Fig. 2).

Altogether, these results reveal a clear difference between amniotes and other vertebrate species. While birds and mammals consistently show a robust co-localization of Runx2 and VDR in cells of the osteoblast lineage, these two proteins do not seem to be co-expressed in amphibian long bones, and the *Runx2 *and *VDR *transcripts only partially overlap in skeletal elements of teleost fishes.

### The teleost Runx2b interacts with the 1α,25-dihydroxyvitamin D3 signaling pathway in mammalian osteoblasts

**Figure 3:**
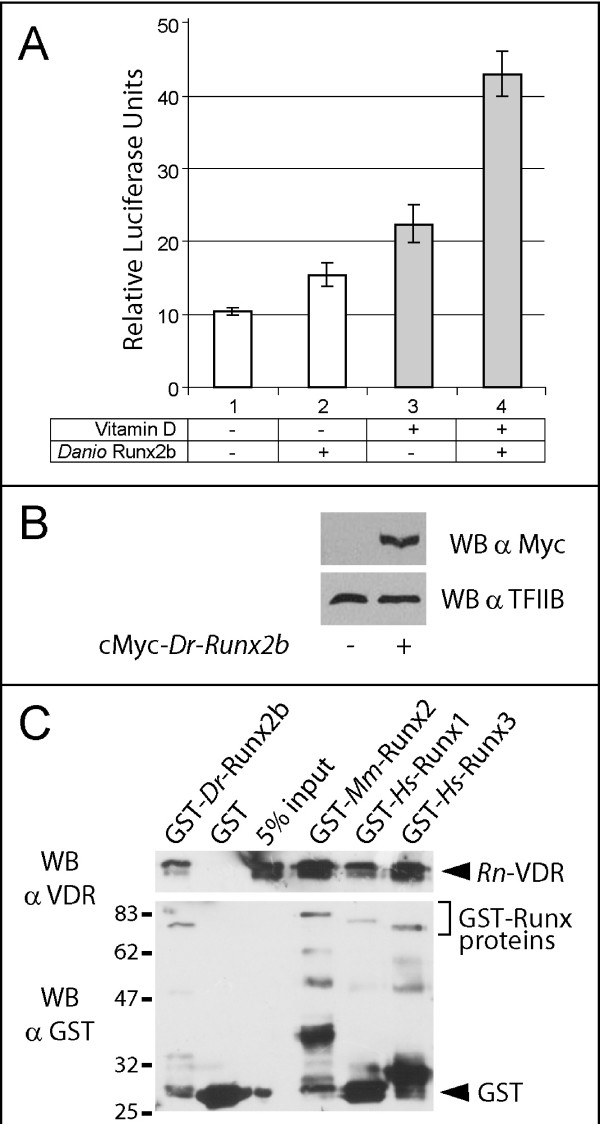
**The teleost Runx2b is functional in mammalian osteoblasts and interacts with the 1α,25-dihydroxyvitamin D3 pathway**. (**A**) ROS17/2.8 osteoblasts were co-transfected for 12 hours with a Myc-tagged version of the *Danio Runx2b *homologue and the promoter of the rat *osteocalcin *driving the expression of the *luciferase *reporter gene. Cells were subsequently incubated for 18 hours in the absence (white bars) or in the presence (grey bars) of 1α,25-dihydroxyvitamin D_3 _(Vitamin D) before being assayed for relative Luciferase activity. (**B**) Western blot performed on nuclear extracts from ROS17/2.8 cells transfected or not with the *Danio Runx2b *homologue. (**C**) Nuclear extracts (150 μg) from ROS 17/2.8 cells cultured in the presence of 10^-8 ^M 1α,25-dihydroxyvitamin D_3 _for 18 h were incubated with GST or with the indicated GST fusion proteins (1.5 μg) previously bound to 20 μl of glutathione-Sepharose beads. Precipitated VDR (upper panel) and GST-Runx (lower panel) proteins were then detected by Western blotting. The migration of molecular weight standards is indicated on the left.

The co-expression of *VDR *and *Runx2 *homologues in some skeletal elements of teleost embryos raises the possibility that *Danio rerio *Runx2 protein can functionally interact with the 1α,25-dihydroxyvitamin D_3 _signaling pathway in differentiating osteoblasts. Hence, we asked if the transcriptional activity of a teleost Runx2 homologue is sensitive to 1α,25-dihydroxyvitamin D_3_. For this purpose, we used cultures of the rat ROS17/2.8 osteoblastic cell line known to increase the expression of osteoblast-specific genes in response to 1α,25-dihydroxyvitamin D_3_. In these cells, the *ocn *promoter directly responds to the mammalian Runx2 protein, and this induction is further increased by the addition of 1α,25-dihydroxyvitamin D_3 _[32]. We found that the overexpression of the *Danio rerio *Runx2b stimulates the expression of a mammalian *ocn *reporter gene (1.5 fold increase), a response that is further enhanced by 1α,25-dihydroxyvitamin D_3 _treatment (2 fold increase, see Fig. 3a, b). The magnitude of this effect was similar to control transfections performed in parallel with the mouse Runx2 homologue (not shown). This functional interaction suggests that the exogenous teleost Runx2b protein can physically interact with VDR-containing macromolecular complexes regulating the transcription of osteoblast-specific target genes. In agreement with this idea, we found that a GST fusion form of the *Danio rerio *Runx2b orthologue is able to interact with the endogenous mammalian VDR obtained from nuclear extracts of immortalized rat osteoblasts (Fig. 3c). Likewise, in this assay, the three mammalian Runx paralogues are able to interact with VDR (Fig. 3c).

### The direct Runx2-VDR interaction is conserved between mammals and teleosts but not between mammals and tunicates

**Figure 4:**
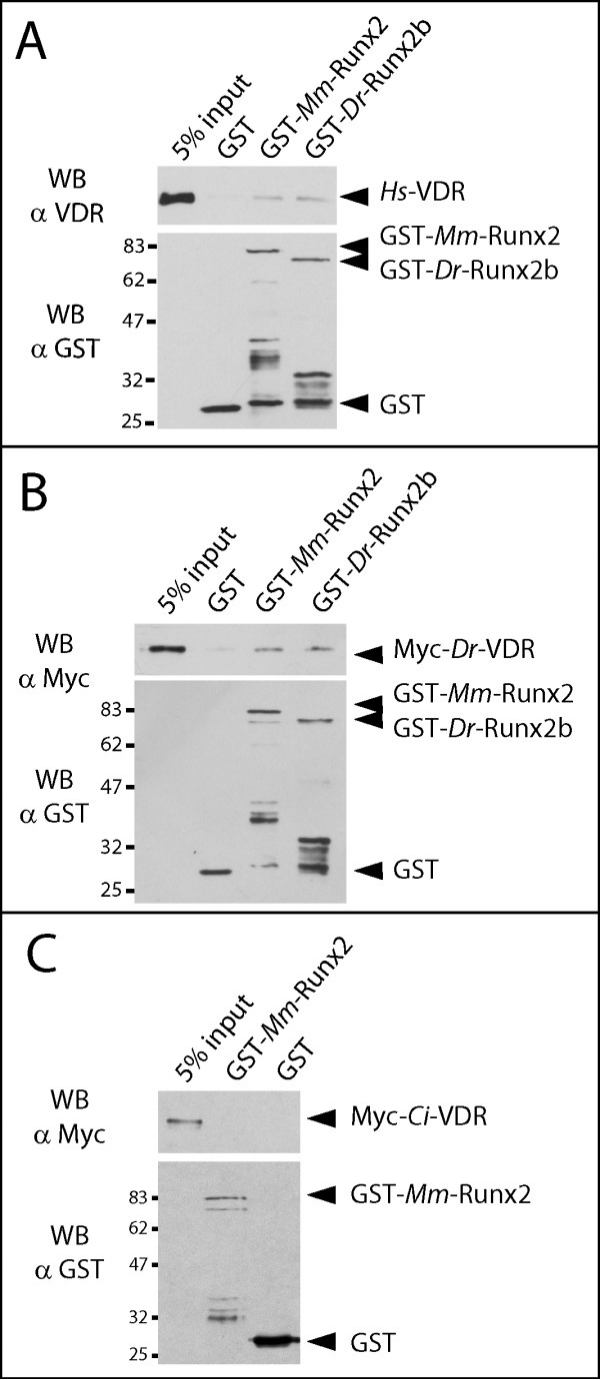
**The Runx-VDR interaction is well conserved between osteichthyan vertebrate species**. GST or the indicated GST-Runx2 fusion proteins (1.5 μg) previously bound to 20 μl of glutathione-Sepharose beads were incubated with the recombinant VDR homologue from *Homo sapiens *(**A**) or *Danio rerio *(**B**). The *Danio rerio *VDR protein is not recognized by the anti-VDR antibody (not shown) and was therefore tagged with an N-terminal Myc epitope. Precipitated VDR (upper panel) and GST-Runx2 (lower panel) proteins were then detected by Western blotting. (**C**) The recombinant VDR homologue from *Ciona intestinalis *(bearing a Myc epitope in its N-terminal) was incubated with GST or the indicated GST fusion proteins (1.5 μg) previously bound to 20 μl of glutathione-Sepharose beads. Precipitated VDR (upper panel) and GST-Runx2 (lower panel) proteins were then detected by Western blotting. Abbreviations: *Dr, Danio rerio*; *Mm, Mus musculus*; *Hs, Homo sapiens*; *Ci, Ciona intestinalis*. The migration of molecular weight standards is indicated on the left.

We subsequently used GST-pull down between purified recombinant proteins to assay the degree of evolutionary conservation of the direct physical interaction previously described between the mammalian Runx2 and VDR [32]. As shown on fig. 4a, the GST-Runx2 proteins from teleosts or mammals can interact, albeit weakly, with the mammalian VDR *in vitro*. We interpret the weak interaction *in vitro *as the requirement of additional cofactors, such as P300 or SRC1, that form macromolecular complexes with the Runx2 and VDR proteins bound to their native target genes [22,32]. Hence, this result supports the idea that the ability of Runx2 to directly interact with a mammalian VDR is well conserved between distantly related osteichthyan vertebrates. To verify if, reciprocally, the teleost VDR protein also bears the ability to directly recognize various Runx2 homologues, we produced a recombinant form of the *Danio rerio *VDR homologue. In GST pull down assays, the *Danio *VDR protein is able to recognize the Runx2 homologues from mammals and teleosts, thereby confirming that the direct Runx2-VDR interaction was possible in the ancestral osteichthyan vertebrate (Fig. 4b). Finally, to assess if the interaction between VDR and Runx homologues is shared with invertebrates, we used an outgroup nuclear receptor from the tunicate *Ciona intestinalis *that is most closely related to the vertebrate VDR [hereafter referred to as Ci-VDR, see [20,21,45]]. *Ci-*VDR is unable to interact with the mammalian GST-Runx2 protein (Fig. 4c). Lowering the stringency of the reaction buffer did not result in any detectable interaction (not shown). Taken together, these findings suggest that the Runx2-VDR interaction is highly conserved between osteichthyan species, but that the invertebrate VDR orthologues are too divergent to be able to interact with vertebrate Runx2 proteins.

## Discussion

With a few recent exceptions, most of our knowledge regarding the genetic basis of osteoblast specification and differentiation comes from experiments performed in mammals [3,11,46-48]. In the present study, we investigated the evolutionary origin of one component of the regulatory network involved in osteogenesis: the functional and physical interaction between the Runx2 and VDR transcription factors [32].

**Figure 5:**
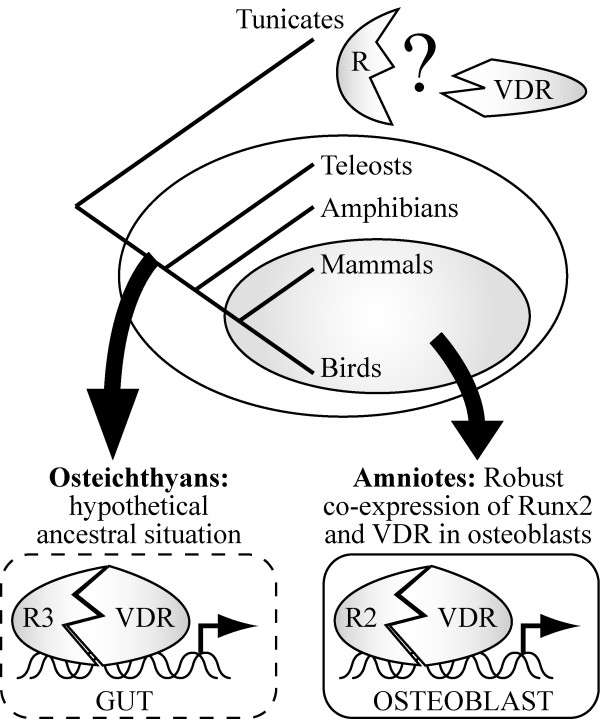
**A model for the evolution of the Runx2-VDR interaction**. A schematic phylogenetic tree showing the evolutionary relationship of invertebrate (tunicates) and vertebrate (osteichthyans) chordates is shown. It is not known if the tunicate Runx (R) and VDR horthologues can interact together (question mark). As Runx3 (R3) and VDR are strongly co-expressed in the gut of many osteichthyan species, their interaction might represent an ancestral, gut-specific, regulatory module. The physical and functional interaction between Runx2 (R2) and VDR probably plays a minor role during osteichthyan skeletogenesis (*i.e. *only in some species or in a subset of skeletal elements). We propose here that it was particularly strengthened in amniotes, once the VDR became robustly expressed in osteoblasts.

Expression analysis revealed a clear difference between amniotes and non-amniote vertebrates. While Runx2 and VDR are robustly co-expressed in nuclei of the osteoblastic lineage of mammals [32] and birds (this study), we found that the two proteins are unlikely to be co-expressed in amphibians and only partially overlap in skeletal elements of teleosts. This observation suggests a relatively recent recruitment of the VDR in the bones of amniotes representative (Fig. 5). Hence, although all vertebrate species possess morphologically similar cells named osteoblasts, important regulatory differences might progressively evolve in specific lineages and modify the transcriptional network controlling the specification and differentiation of this cell type. From our results it is tempting to propose that the VDR plays a direct, cell autonomous, role in all osteoblasts of amniotes, but not in amphibians (Fig. 5). In *Danio rerio*, 1α,25-dihydroxyvitamin D3 increases mineralization [49]. Our results suggest that this hormone regulates bone mineralization indirectly (via the control of calcium and phosphate homeostasis in intestinal cells), but also cell-autonomously, in the osteoblasts of the specific skeletal elements where Runx2 and VDR are co-expressed (*e.g. *the parasphenoid and the operculum). A broader sampling of actinopterygian, sacrcopterygian as well as chondrichthyan species would provide valuable information regarding this issue.

Using *in vitro *GST pull-down assays, we observed that the mammalian and teleost VDR proteins can interact with various Runx homologues. In particular, the interaction is conserved with the two mammalian paralogues (Runx1 and Runx3) and with the teleost orthologue (*Danio rerio *Runx2). These experiments reveal that the interaction domains between the ancestral VDR and Runx proteins were already compatible in the last common ancestor of all osteichthyan vertebrates, and have remained well conserved. In agreement with these data, we confirmed that the Runx2b protein from teleosts is functional in mammalian osteoblastic cells and can be further stimulated by the addition of the VDR ligand. These results suggest that this hormone can stabilize Runx2-VDR complexes on the promoter of specific target genes in teleost osteoblasts.

We failed to detect an interaction between the VDR homologue from tunicates and a vertebrate Runx2 orthologue. In this regard, it is relevant to point out that the *Ci*-VDR transcription factor differs from its vertebrate homologues in many other respects, as it is unable to activate reporter genes and to interact with 1α,25-dihydroxyvitamin D_3 _[21,45,50,51]. Hence, after the split from tunicates, the ancestral vertebrate VDR underwent dramatic structural modifications, both in the C-terminal ligand-binding domain [21,51] and in the Runx2-binding domain mapped to the N-terminal region [32]. It is possible, but unproven, that the Runx and VDR orthologues are undergoing a constant co-evolutionary process and interact together in tunicates (Fig. 5).

In sharp contrast with the highly conserved skeleton-specific expression of *Runx2*, the transcripts and protein product of the *VDR *gene are detected in a broad variety of lamprey, teleost and amphibian adult organs [37,38,45,50]. One might stipulate that the functional cooperation observed between VDR and Runx2 can be extended to the other vertebrate Runx paralogues that are co-expressed with the VDR. Therefore, in addition to osteoblasts, it is possible that 1α,25-dihydroxyvitamin D_3 _signaling directly integrates the Runx-dependent regulatory networks involved in the specification of many vertebrate tissues where VDR is co-expressed with Runx1 or Runx3. Indeed, the fairly ubiquitous expression of the VDR might have increased its chances of being co-expressed with (and to interact with) any of the three Runx paralogues. In this respect, it is interesting to correlate the expression of Runx and VDR homologues in endodermal tissues. On the one hand, *Runx *orthologues of nematode, sea urchin, amphioxus and mammals (*Runx3*) are expressed in the gut [52-55], suggesting an ancient endodermal expression inherited from the urbilateria. On the other hand, *VDR *is expressed in the intestine of teleosts [37], lampreys [45], frogs [50], birds [56] and mammals [57]. It is tempting to propose that shortly after its emergence, the VDR protein was co-expressed in the gut with Runx3 (Fig. 5). Once the first Runx-VDR regulatory interaction established, it would have been free to spread to the other Runx transcription factors and their tissue-specific target genes by exaptation and regulatory rewiring [58,59].

## Conclusions

In summary, we propose that the molecular machinery involving Runx2 and VDR in osteoblastic cells exists at least since the emergence of the osteichthyans, but has subsequently been strengthened in the lineage leading to amniotes. The widespread VDR distribution might have facilitated the co-expression with the Runx paralogues, and contributed to the emergence of the Runx-VDR physical and functional interaction in a variety of tissues. Osteoblasts from distantly-related vertebrate species secrete mineralized matrix and exhibit a similar morphology [60]. Yet, in spite of these shared characters, genomic turnover continuously creates, eliminates and modifies genes coding for bone matrix proteins, a phenomenon called phenogenetic drift [61]. Our results suggest that, likewise, the osteoblast-specific regulatory network has steadily been evolving during the vertebrate radiations. Although the essential Runx2-dependent regulatory kernel has remained highly conserved, it should not come as a surprise if, between species, osteoblastogenesis is modulated by a plethora of different transcription factors, signaling pathways, and ligands, such as the 1α,25-dihydroxyvitamin D3.

## Methods

### Immunohistochemistry

*Gallus gallus *E7 and *Xenopus tropicalis *stage 58 [62] hindlimbs were dissected out and fixed for three days in Bouin (14 volumes of picric acid, 5 volumes of formaldehyde 37% and 1 volume of glacial acetic acid). Samples were subsequently embedded in paraffin, sectioned at a seven micrometer thickness, mounted on glass slides and stained following a classical hematoxylin eosin procedure. For immunohistochemistry, hindlimbs from *Gallus gallus *E7 embryos and from *Xenopus tropicalis *stage 58 tadpoles were removed, mounted in OCT (Sakura Finetek, Torrance, CA, USA) and quickly frozen in isopentane cooled with liquid nitrogen, as described [63]. Cryosections (20 micrometer) were immunostained with primary antibodies diluted 1:100 in blocking solution (1% BSA in Dulbecco's phosphate buffer saline; D-PBS) 12-15 h at 4°C. Antibodies were C-20 (rabbit polyclonal anti-rat VDR, Santa Cruz Biotechnology) and a mouse monoclonal anti-Runx2, kindly provided by the laboratory of Dr. Gary Stein. Control experiments performed in the absence of primary antibodies gave negative results (not shown). Corresponding alexa488 and alexa546-conjugated secondary immunoglobulins (Invitrogen) were incubated for 2 h at RT. Nuclei cells were counterstained with TO-PRO-3 iodide (Invitrogen). Samples were subsequently mounted with aqueous medium for fluorescence (Sigma). Images were acquired with a laser confocal Nikon Eclipse TE2000-U microscope.

### *In situ* hybridization and RT-PCR on *Danio rerio* embryos

*Danio rerio *embryos were raised at 28°C and fixed for *in situ *hybridization in 4% paraformaldehyde. Hybridization reactions were performed as previously described [64]. The *Danio rerio VDR *probe covered the region coding for the ligand binding domain [65]. Embryos were mounted in glycerol, observed under a Leica MZ12.5 stereomicroscope and photographs were taken with a Leica DC300F digital camera.

The mRNA for expression studies was extracted from embryos or larvae at different stages of development (0, 24, 48 and 72 hpf) using the Trizol Reagent according to the manufacturer's indications (Invitrogen). Reverse transcription was performed with the SuperScript II (Invitrogen) according to the manufacturers' instructions. As an internal control, we used β-actin primers: Forward 5'-TTC TGG TCG GTA CTA CTG GTA TTG TG-3' and reverse 5'-ATC TTC ATC AGG TA- GTC TGT CAG GT-3'. The sequences of the *VDR *primers were as follow: Forward 5'-TCA CTG ATG GAT CTG ATG GC-3' and reverse 5'-CTG AAT CTG ACG AAG TCG GA-3'.

### Transfection and luciferase assay

The rat ROS 17/2.8 osteoblastic cells were cultured as described previously [32]. Cells were plated in 24-well plates and transiently transfected with the *Rattus norgevicus osteocalcin*-*luciferase *reporter (pOC-LUC, 50 ng/well), the renilla internal control (pSV40-*renilla*, 2.5 ng/well) and a Myc tagged version of the full length *Danio rerio *Runx2 open reading frame under the control of the CMV promoter (100 ng/well). The total amount of transfected DNA was maintained at 650 ng/well with pBluescript. ROS 17/2.8 cells were transfected with Lipofectamine Plus reagent (Invitrogen) according to the manufacturer's instructions. Six hours after transfection, 1α,25-dihydroxyvitamin D_3 _was added to the medium at a final concentration of 10^-8 ^M. Cells were harvested 24 h after transfection and assayed for Luciferase and Renilla activity using the Luciferase Assay System (Promega) in a TD20/20 luminometer (Turner Designs). The efficiency of the overexpression was verified by Western blots on nuclear extracts prepared from transfected cells.

### GST-pull down assays

The proteins containing the N-terminal glutathione S-transferase (GST) fused in frame to the Runx homologues were obtained by expression in *Escherichia coli *BL21 strain as previously reported [32]. GST-free proteins were obtained by cleaving GST-VDR or GST-Runx2 orthologues with 25 U of Thrombin (Amersham Biosciences) at 4°C overnight. Nuclear extracts were prepared from 15 plates of confluent ROS 17/8.2 previously treated with 10^-8 ^M 1α,25-dihydroxyvitamin D_3 _for 18 h. The plates were placed on ice for 10 min, and then washed with 10 ml of cold PBS. Cells were collected with a scrapper in 15 ml of cold PBS with Complete protease inhibitor Cocktail (Roche), and centrifuged at 2000 rpm for 5 min at 4°C. The Cells were resuspended and incubated on ice for 5 min in 5 volumes of pellet equivalent of buffer A (10 mM HEPES pH7,9; 1,5 mM MgCl2; 10 mM KCl; 1 mM DTT and 1× Protease inhibitor). Cells were lysed using a Dounce homogenizer and centrifuged at 3000 rpm for 15 min at 4°C. Pelleted nuclei were washed with 5 ml of cold buffer A, centrifuged at 12 000 rpm for 10 min at 4°C, resuspended in 100 μL of cold buffer C (20 mM HEPES pH7,9; 1,5 mM MgCl2; 420 mM KCl; 0,2 mM EDTA; 1 mM DTT; 1× Complete Protease inhibitor), and incubated for 1 h at 4°C with gentle agitation. After centrifuging at 12 500 rpm for 15 min at 4°C the supernatant (nuclear extracts) was collected, quantified using a classical Bradford assay, and rapidly frozen with 25% glycerol. The GST-pull down assays were performed with 25 μl of Glutation Sepharose resin (Pharmacia Biotechnologies). 1 μg of GST, Runx2-GST (or VDR-GST) fusion proteins were incubated in 400 μl of binding buffer (20 mM Tris pH 8.0, 100 mM KCl, 0.5% NP-40, 10 mM EDTA, 0.05 mM PMSF, 1 mM DTT) for 30 min at 4°C with gentle agitation. 1.5 μg of pure recombinant VDR (or Runx2) proteins were added in a final volume of 500 μL of the binding buffer containing 0.5% of non-fat milk, and were subsequently incubated for 2 h at 4°C with gentle agitation. The reactions were washed four times with 500 μL binding buffer for 5 min at 4°C. After the last centrifugation, the resin was resuspended in loading buffer (100 mM Tris-Cl pH 6.8, 4% p/v SDS, 0.15% v/v bromofenol blue, 20% v/v glycerol, 200 mM DTT) and incubated for 5 min at 95°C. The proteins that were retained with the resin were run in a 10% acrylamide SDS-PAGE and revealed by Western blotting with specific antibodies against VDR (C-20, Santa Cruz Biotechnology), GST (Pharmacia Biotech) and cMyc (9E10, Santa Cruz Biotechnology). The accession numbers of the cDNAs coding for the proteins used in this study are: *Homo sapiens Runx1 *(NM_001754), *Mus musculus Runx2 *(NM_004348), *Homo sapiens Runx3 *(NM_004350.2), *Danio rerio Runx2 *(AY443097), *Homo sapiens VDR *(NM_000376) and *Ciona intestinalis VDR-PXR *homologue (AB210742). The *Danio rerio *full length *VDR *clone was reconstituted by PCR using overlapping 5' and 3' regions as templates. The 3' region corresponded to the ligand binding domain [described in [65]]. The 5' region coding for the DNA binding domain was obtained by RT-PCR using 72 hpf cDNA as a template (forward primer 5'-TCA CTG ATG GAT CTG ATG GC-3' and reverse primer 5'-CTG AAT CTG ACG AAG TCG GA-3').

## Authors' contributions

SM carried out pull down experiments with nuclear extracts, participated to transfection experiments and to immunohistochemical staining of chick and frog long bones, and wrote the paper. SM and MM designed the study and analyzed the data. CB carried out pull down experiments with recombinant proteins. BH participated in the cell cultures and in transfection experiments. JPH and MA participated in the immunohistochemical staining of chick and frog long bones. AER and EHB performed the RT-PCR and *in situ *hybridization experiments with *Danio rerio*. All authors read and approved the final manuscript.
